# Multi-omics assessment of gut microbiota in circadian rhythm disorders: a cross-sectional clinical study

**DOI:** 10.3389/fcimb.2025.1524987

**Published:** 2025-03-27

**Authors:** Yuting Tian, Rong Zhao, Shili Xiao, Lu Chen, Yi Cheng, Wei Meng, Zongyuan Tang, Yi Cai, Zhifeng Xiao, Ailin Yi, Minjia Chen, Xuefei Zhao, Guangcong Ruan, Yanling Wei

**Affiliations:** Department of Gastroenterology, Chongqing Key Laboratory of Digestive Malignancies, Daping Hospital, Army Medical University (Third Military Medical University), Chongqing, China

**Keywords:** gut microbiota, circadian rhythm disorder, metabolites, cross-sectional study, multiomics analysis

## Abstract

**Background:**

The interaction between the host and microbiota is influenced by host circadian rhythm. However, it is unknown what the changes of gut microbiota and metabolites.

**Methods:**

We conducted a cross-sectional study (n=72) in which participants’ fecal DNA was detected by macrogenomic sequencing analysis. The feces, urine and blood were analyzed by widely targeted metabolomics analysis.

**Results:**

Pearson correlation analysis showed that most of the clinical symptoms of people with circadian rhythm disorders were moderately positively correlated with gastrointestinal symptoms. By distilling the results of multinomic analysis, we reported a variety of different species (19 species in the gut) and metabolites. In our results, the correlation of multiomics is mostly concentrated in *Lachnospiraceae bacterium* and *Streptococcus mitis oralis pneumoniae*. Bile acid-related metabolites are the most significant metabolites associated with these species.

**Discussion:**

Our study demonstrates the severity of clinical manifestations caused by circadian rhythm disorder is closely related to microbiota and metabolism. In the future, personalized interventions targeting specific microbial species or metabolites may help alleviate the physical and psychological discomfort induced by circadian rhythm disturbances.

## 1 Introduction

Circadian rhythms are 24-hour patterns regulating behavior, organs, and cells in living organisms. These rhythms align biological functions with regular and predictable environmental patterns to optimize function and health. Disruption of the circadian rhythm will lead to rhythm disorders, which may be harmful to our bodies (Meyer et al., 2022). As a result of disrupted circadian rhythms, people working night shifts inevitably lead to fatigue and sleep disturbances, that increase the risk of harmful health outcomes such as gastrointestinal disorders, cardiovascular diseases, mental disorders (depression or anxiety), injuries, and musculoskeletal pain (Neves et al., 2022).

Recent observations of interactions between gut microbiota and host circadian rhythm raise an intriguing hypothesis that many of adverse effects of clock disruption may be due to, in part, to the impact of an altered diversity and/or composition of gut microbiota (Kramer et al., 2022; Zhang et al., 2023b). Together, gut microbiome is one of the key factors that is involved in maintaining host circadian rhythms. Persistent jet lag, an obesogenic diet, and clock gene deficiency can affect the composition and metabolism of gut microbiota. Gut microbiota dysbiosis reduces the production of metabolites, including short-chain fatty acids and bile acids. Gut microbial metabolites, such as short-chain fatty acids (Zhang et al., 2023a), can not only affect intestinal barrier function, inflammation, oxidative stress, and colonic carcinogenesis (Liu et al., 2021b) but also affect clock gene expression and decouple the peripheral clock from the master clock, thus changing the amplitude of circadian genes (Fawad et al., 2022). Some reports on mice have shown that disorders of clock genes in mice could lead to the aggravation of DSS-induced enteritis (Hiramoto et al., 2018; Liu et al., 2021a). The damage and inflammation of the intestinal barrier can also lead to damage to cognitive function in mice (Meng et al., 2022). On the other hand, the disorder of gut microbiota caused by circadian rhythm disorder can disrupt the host’s metabolism, energy homeostasis, and inflammatory pathways, leading to metabolic syndrome, inflammation, and cancer (Bishehsari et al., 2020; Razi Soofiyani et al., 2021; Fang et al., 2023; Yang et al., 2023).

The complex interplay between circadian rhythm disorders and gut microbiota has received significant attention in recent years. A growing body of research has demonstrated that gut microbiota, along with other interventions such as diet, can modulate the host’s circadian rhythm (Zheng et al., 2020; Gutierrez Lopez et al., 2021) and influence daytime cognitive and metabolic functions (Li et al., 2018; Codoñer-Franch et al., 2023). However, current studies have primarily focused on single-omics analyses, lacking a systematic exploration of the multidimensional relationships among gut microbiota, metabolites, and host health status. Therefore, integrated multi-omics analysis offers substantial advantages for in-depth investigation of the intricate interactions between circadian rhythm disorders, its related health outcomes, and gut microbiota. Furthermore, this multidimensional approach is expected to provide a more comprehensive understanding of how gut microbiota responds to and influences the host’s circadian clock. In this cross-sectional study, we aim to explore the relationships between health status, gut microbiota composition, and metabolic profiles in individuals with circadian rhythm disorders, thereby laying the groundwork for future in-depth research.

Our results showed that participants with circadian rhythm disorders had poorer sleep quality, fatigue, anxiety, and depression and more obvious gastrointestinal symptoms than the healthy participants. There were differences in the composition of gut microbiota between participants with circadian rhythm disorders and healthy controls, in metabolites in blood, feces, and urine. The correlation analysis of metagenomics, metabolomics, and questionnaire survey results showed that the composition of gut microbiota and the destruction of metabolic balance were closely related to the clinical symptoms caused by circadian rhythm disorders.

## 2 Materials and methods

### 2.1 Subject participation

**Figure 1:**
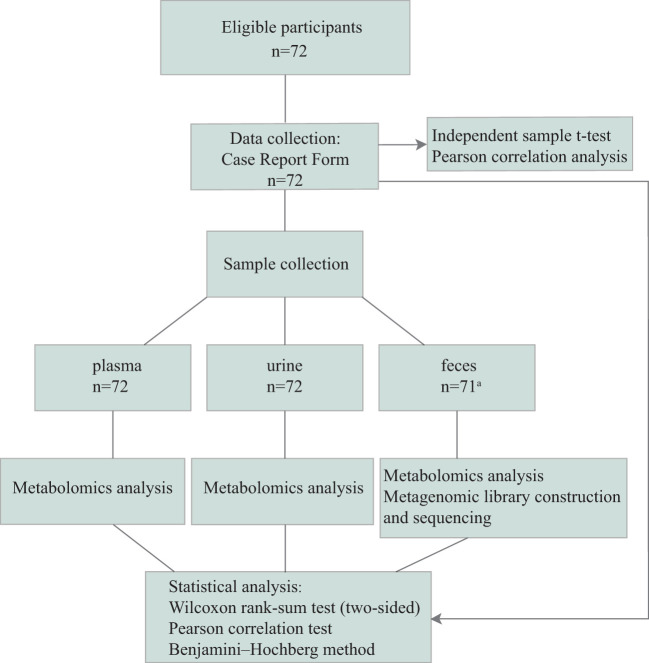
Consort. The case report form (CRF) contains: Basic information of participants; Circadian Type Inventory (CTI); Gastrointestinal Symptom Rating Scale (GSRS); Epworth Sleeping Scale (ESS); Fatigue Scale-14 (FS-14); Maslach Burnout Inventory-General Survey (MBI-GS); Athens Insomnia Scale (AIS); and Depression Anxiety Stress Scales-21 (DASS-21). ^a^one stool sample was not collected on time.

Thirty-six circadian rhythm disorders (CRD) and 36 healthy controls (**Figure 1**) from the Army Medical Center of PLA between January 2022 and December 2022 were recruited. All participants provided written informed consent before participation.

### 2.2 Data collection

The primary outcome was the change in gut microbiota in fecal samples. The secondary outcomes included the changes in metabolites of gut microbiota in fecal samples; the changes in plasma metabolites; the changes in urine metabolites; Circadian Type Inventory (CTI); Gastrointestinal Symptom Rating Scale (GSRS); Epworth Sleeping Scale (ESS); Fatigue Scale-14 (FS-14); Maslach Burnout Inventory-General Survey (MBI-GS); Athens Insomnia Scale (AIS); and Depression Anxiety Stress Scales-21 (DASS-21).

Notably, circadian rhythm disorders are harmful. Here, we collected the sleep status, mental status, and gastrointestinal symptoms of CRD. Lack of sleep and circadian rhythm disorder will lead to poor mental state and metabolism (Chaput et al., 2022). The homeostatic drive for sleep and rhythm of sleepiness often lead to body fatigue, which greatly affects people’s work and life (Caldwell et al., 2019). At the same time, there is also a certain link between sleep status and depression, and it seems that circadian rhythm disorder also plays an important role in it (Pandi-Perumal et al., 2020). The simple assessment of gastrointestinal symptoms is evidence used to explore the changes in body state caused by circadian rhythm disorder.

All participants were selected to collect questionnaire information, fecal samples, and fasting blood and urine samples. The time point was collected after the night shift work of the circadian rhythm group, and the healthy controls were collected in the morning of the normal working day. Participants reported their CTI for nearly a month. The CTI includes two subscales, of which the Flexibility/Rigidity scale (FR) has five items. The higher the score, the stronger the flexibility of the rhythm. Languid/vigorous (LV) has six items, and the higher the score, the more serious the fatigue of the human body. The GSRS was measured to comprehensively assess common gastrointestinal symptoms for nearly a week. The higher the score, the more serious the gastrointestinal symptoms. ESS is a widely used tool validated to measure sleepiness. The higher the score, the more serious the sleepiness symptoms. FS-14 was used to evaluate the severity of fatigue. The higher the score, the heavier the fatigue symptoms. The AIS is a self-assessment psychometric instrument designed for quantifying sleep difficulty based on the International Classification of Diseases-10 criteria. The higher the score, the more serious the insomnia. The MBI-GS is a scale widely used in the general occupation to measure job burnout. After scoring more than 50 points, the higher the score, the less positive the work attitude. The DASS-21 can validly be used to measure the dimensions of depression, anxiety, and stress. The higher the score, the worse the mental state.

### 2.3 Sample collection and assessment

Fresh fecal samples were collected from participants on the day of the visit, and approximately 30-50 g was sampled in a sterile container and stored at -80°C immediately. Blood and urine samples were collected from the same participants at the same visit. Blood samples were collected to stand at room temperature for 1 h to ensure complete clotting. Plasma was obtained by centrifugation at 3,000 rpm for 15 min at 4°C and stored at −80°C for metabolomics.

Frozen fecal samples were delivered to the BGI-Wuhan Sequencing Service Center for microbial metagenomic library construction and sequencing. Briefly, microbial DNA was extracted from the fecal samples using the MagPure Stool DNA KF kit B (Magen, China), and the metagenomic libraries were prepared using the MGIEasy Fast FS DNA Library Prep Set (MGI, China) according to the manufacturer’s protocols. PE150 sequencing was performed using the MGI DEBSEQ2000 platform.

Fecal, plasma, and urine samples saved for metabolite analyses were delivered to METWARE BIO (Wuhan, China) for ‘TM’ widely targeted metabolomics analysis as previously described (Chen et al., 2013).

### 2.4 Metagenomic sequencing analysis

The PE150 reads from MGI DEBSEQ2000 were obtained and processed using our in-house pipeline consisting of the following tools: fastp v0.20 (Chen et al., 2018) was used to trim the sequencing and PCR adapters and filter short sequences (<50 bp), followed by kneaddata v0.7.4 (McIver et al., 2018) to trim the low-quality reads (a sliding window of 4 and average quality score of 20 was used) and decontaminate against the hg19 human genome; (Franzosa et al., 2018) the HMP Unified Metabolic Analysis Network 2.0 (HUMAnN 2.0) was used to generate taxonomy profiles and functional profiles. Alpha diversities based on the taxonomy profile were obtained in the form of metagenomic species count and Shannon index using vegan v2.6.4. Beta diversities of taxonomy or functional profiles were obtained using the Bray−Curtis distance, and PCoA was performed using the cmdscale function in vegan v2.6.4.

### 2.5 Metabolomics analysis

Relative concentrations in the form of peak areas from a rich set of metabolites can be obtained from the TM-targeted metabolomics analysis. The obtained metabolites were first filtered to remove any entry with >15% relative standard deviation (RSD, standard deviation/mean) among the 8 QC samples since these metabolites cannot be reliably measured. Missing values were then imputed with ½ of minimal peak area for each metabolite across all samples. Finally, each metabolite was normalized by the total peak areas of all metabolites in the respective samples, followed by log10 transformation and Pareto scaling. The processed metabolite measurements were fed into downstream analyses.

### 2.6 Quantification and statistical analysis

For analysis by Characteristics of patients, the independent sample t-test was used for comparison between two groups, and Pearson correlation analysis was used for correlation analysis. The Shapiro-Wilk test was used to evaluate whether the continuous data conformed to a normal distribution. The differential abundance of species, MetaCyc pathways and metabolites between the two groups was tested by the Wilcoxon rank-sum test (two-sided) via the R build-in function Wilcoxon test. Correlations between continuous variables, including species abundance, metabolite abundance and clinical characteristics, were analyzed by using the Pearson correlation test. All analyses and results visualization were performed in R V4.3.1 with R packages. For the independent sample t-test, the mean differences between groups were reported with 95% confidence intervals (95% CI). For the Pearson correlation test, the correlation coefficients (r) were reported with 95% confidence intervals (95% CI). The p-values of multiple testing was corrected as the false discovery rate (FDR) with the Benjamini-Hochberg method and a p-value or FDR-corrected p-value <0.05 was considered statistically significant.

## 3 Results

### 3.1 Circadian rhythm disorders were linked to heightened fatigue, insomnia, and gastroenterology symptoms

After organizing the scales and data collected in the cross-sectional study, we found considerable differences between the two groups. The 72 volunteers we selected were divided into two groups (**Figure 1**). The p-values of age (p=0.173, 95%CI:−4.62 to 0.85) and body mass index (BMI, p=0.590, 95%CI:−1.08 to 1.88) of the two groups were greater than 0.05 (**Table 1**), indicating that age and BMI did not affect the differences between the two groups. For other questionnaire data (GSRS/ESS, etc.) collected, we calculated the p-value by t-test and found that there was a difference (FR was not included) between the two groups.

**Table 1:** Characteristics of participants in circadian rhythm disorders and healthy controls.

		Healthy controls	CRD	p-value
Age (y)		27.11 (6.61)	29.00 (4.88)	0.173
BMI (Kg/m^2^)		22.37 (2.18)	21.97 (3.85)	0.590
CTI	FR	14.22 (4.45)	15.81 (3.77)	0.108
	LV	21.17 (3.50)	23.61 (3.37)	0.004^a^
	total	35.39 (5.69)	39.31 (5.93)	0.006^a^
GSRS		22.33 (7.16)	31.50 (9.85)	<0.001^a^
ESS		7.58 (3.20)	9.72 (3.73)	0.011^a^
FS-14	Physical fatigue	4.17 (2.10)	5.69 (1.97)	0.002^a^
	Mental fatigue	1.17 (1.25)	3.31 (1.56)	<0.001^a^
	total	5.33 (3.00)	9.00 (3.23)	<0.001^a^
AIS		3.58 (2.78)	8.03 (3.68)	<0.001^a^
MBI-GS		36.90 (16.11)	50.17 (17.13)	0.001^a^
DASS-21	Depression	2.03 (2.35)	4.03 (3.72)	0.008^a^
	Anxiety	2.11 (2.23)	4.22 (3.19)	0.002^a^
	Stress	3.06 (2.99)	5.42 (3.43)	0.003^a^

The values in Table indicates average value (SD) for continuous variables. Using the t-test, p-values were calculated to assess differences between circadian rhythm disorders (CRD) and healthy controls. Body fat mass index is calculated as body fat (kg)/height (m^2^). Flexibility/Rigidity scale (FR), Languid/vigorous (LV).

a Significant p-value (p<0.05).

The results of the clinical data demonstrated differences between the two groups showing that the ability of participants with circadian rhythm disorders to overcome drowsiness (ESS) is challenging. They are also more prone to fatigue and insomnia, and their mental and work status are also worse (The mean[SD] value of CRD was higher than that of healthy control group). In addition, the overall score of gastrointestinal symptoms (Mean[SD], 31.5[9.85]) of participants with circadian rhythm disorder is higher, and the symptoms are more serious. Inspired by the significant change in clinical data, we investigated the correlation between GSRS and other scales. Pearson correlation analysis (**Table 2**) showed that there was a linear relationship between some scales (FR, ESS and physical fatigue was excluded) and GSRS in participants with circadian rhythm disorders. There was a moderate positive correlation with GSRS (r>0.300, p<0.05), including fatigue, insomnia, mental state, and other data. Significantly, the probability value of ESS (p=0.011, 95%CI:−3.77 to −0.50) in **Table 1** was higher than AIS score (p<0.001, 95%CI:−5.98 to −2.91).

**Table 2:** Pearson correlations between GSRS and other clinical data in circadian rhythm disorders.

		Pearson correlation (r)	p-value
CTI	FR	0.223	0.192
	LV	0.342	0.041^a^
	total	0.343	0.041^a^
ESS		0.243	0.153
FS-14	Physical fatigue	0.294	0.082
	Mental fatigue	0.491	0.002^a^
	total	0.416	0.012^a^
AIS		0.436	0.008^a^
MBI-GS		0.586	<0.001^a^
DASS-21	Depression	0.548	0.001^a^
	Anxiety	0.513	0.001^a^
	Stress	0.631	<0.001^a^

Pearson correlation(r) coefficients are reported.

a Significant p-value (p<0.05).

To explore the relationship between the disordered circadian rhythm and gut microbiota, we collected stool, plasma, and urine samples to further analyze the changes in intestinal microorganisms and the metabolome.

### 3.2 Microbiome alterations between participants with circadian rhythm disorders and healthy controls

**Figure 2:**
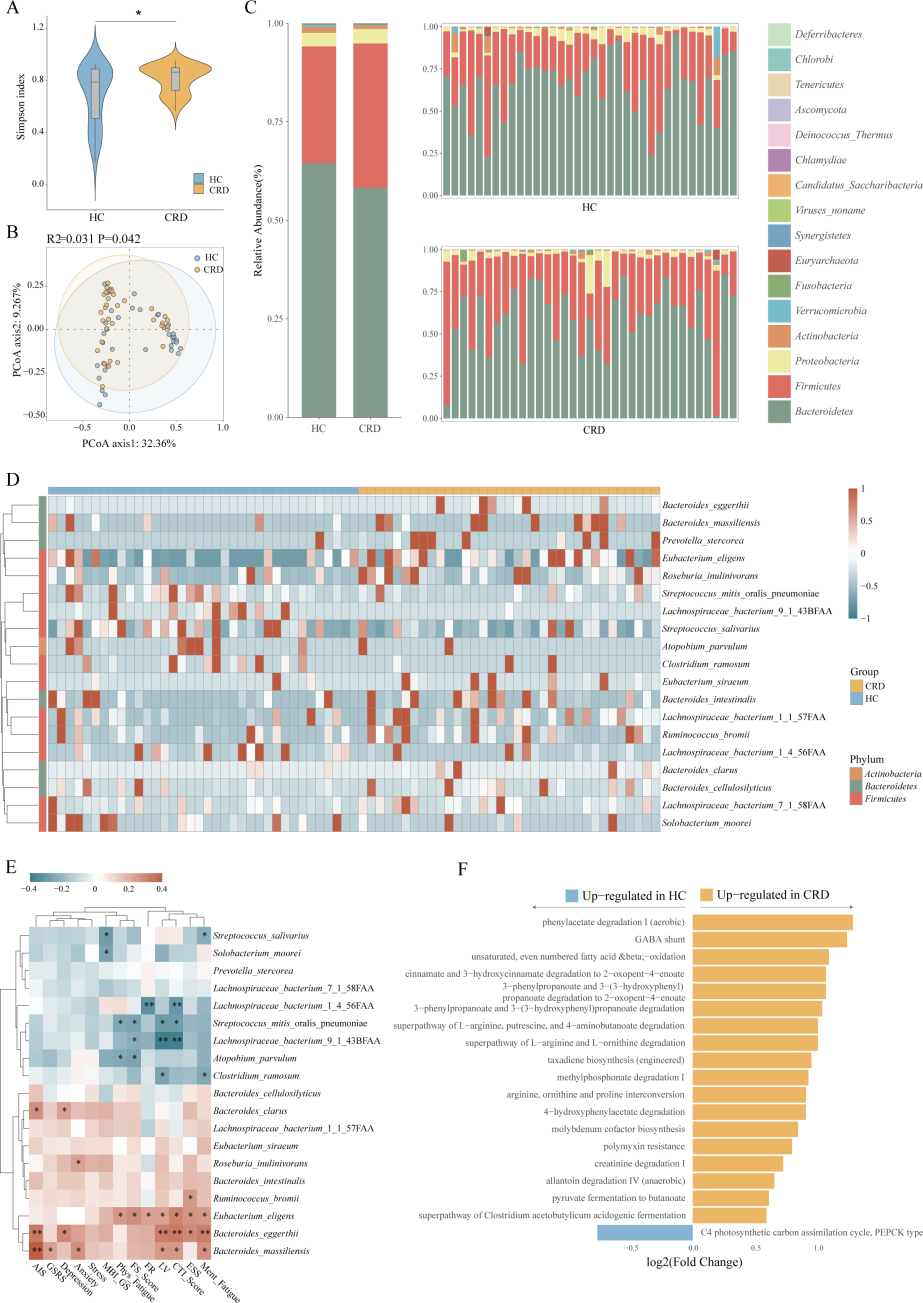
Gut microbiota alterations between CRD and healthy controls. **(A)** α-diversity expressed as species diversity for CRD and healthy controls. **(B)** Principal coordinates analysis based on unweighted UniFrac showing the distribution along principal component (PCo) 1 and 2 of CRD and healthy controls samples. The numbers next to the axis indicate the amount of compositional variation explained by each PCo. **(C)** Differentially abundant gut microbiota species at the phylum level. Figure **C** shows the overall differential distribution on the left and the individual differential distribution of gut microbiota on the right. **(D)** The relative abundance of 19 dominant genera in the gut microbiota between CRD and healthy controls. **(E)** The heatmap shows the associations between 19 differentially abundant species and 13 kinds of questionnaire survey results. **(F)** The X-axis represents the relative abundance of MetaCyc pathway, while the Y-axis displays differential metabolic pathways in participants with circadian rhythm disorders. Circadian rhythm disorders (CRD) and healthy controls (HC).

We collected a total of 72 blood samples, 72 urine samples, and 71 fecal samples from 36 non-shift workers and 36 day and night shift workers (one stool sample was not collected on time). To analyze the gut microbiota of participants with circadian rhythm disorder, we performed metagenomics detection on all collected fecal samples. The Simpson index (**Figure 2A**) showed that there were differences in the diversity of microbiota between the two groups. The principal coordinate analysis (PCoA) diagram (**Figure 2B**) shows that the icons of participants with circadian rhythm disorder and the healthy control group are clustered together, which indicates that fecal metagenomic data may be weak in distinguishing whether the circadian rhythm is disordered. In agreement with the findings regarding overall microbiota composition, we found differential abundance (**Figure 2C**) of dominant phylum in the fecal microbiota between CRD and healthy control samples. Some phyla decreased in CRD, including *Bacteroidetes*, *Actinobacteria*, *Verrucomicrobia*, and so on. The change of *Bacteroidetes* was the most obvious. Among the increased phyla (*Firmicutes*, *Fusobacteria*, *Euryarchaeota*, etc) *Firmicutes* showed the greatest change. The changes of *Bacteroidetes* and *Firmicutes* together constitute the most pronounced difference in participants with circadian rhythm disorder.

By analyzing the gut metagenomic profiles of 71 samples from the discovery cohort, we identified 19 differentially abundant gut microbiota genera (**Figure 2D**), of which 12 were elevated in the CRD relative to the healthy controls, including *Bacteroides cellulosilyticus*, *Eubacterium siraeum*, *Eubacterium eligens*, *Roseburia inulinivorans*, *Prevotella stercorea*, *Bacteroides massiliensis*, *Lachnospiraceae bacterium*(*L. bacterium*) 1 1 57FAA, etc. These genera could be specific for the CRD microbiota. In comparison, only seven species were enriched in the healthy group, including two species from the genus *L. bacterium*, *Streptococcus mitis oralis pneumoniae*, *Clostridium ramosum*, and others. The enrichment of *L. bacterium* 9 1 43BFAA in the healthy group was the most significant, followed by *L. bacterium* 1 4 56FAA.

We collected 12 questionnaire factors from 72 volunteers for correlation analysis with their different gut microbiota. The results showed that among the questionnaire factors, 12 questionnaires had 37 associations with 19 different gut microbiota (**Figure 2E**). There were 15 kinds of negative correlations and 22 kinds of positive correlations. Simply categorizing these scales, the 6 questionnaire items about fatigue (CTI/MBI-GS/FS-14) contain a total of 14 negative correlations and 12 positive correlations. The two questionnaire items about sleep (AIS/ESS) contained 0 negative correlations and 6 positive correlations. The three questionnaire items related to emotion (DASS) contained 0 negative correlations and 4 positive correlations. One questionnaire item (GSRS) about the gastrointestinal tract contained 0 negative correlations and 1 positive correlation. Through simple classification, we can observe that there are both positive and negative correlations in the questionnaire data related to microbiota and fatigue. In other data (sleep/mood/intestinal tract), the difference correlation showed a positive correlation. The impact of gut microbiota on fatigue-related questionnaire data is balanced, but the impact on sleep, emotion and other issues is biased.

We found that if the microbiota is the main body of the heatmap, the characteristics of correlation aggregation are obvious. *Eubacterium Eligens*, *Bacteroides Eggerthii*, *Bacteroides Massiliensis* and other species (these species are enriched in participants with circadian rhythm disorders.) are positively correlated with our clinical data, *L. bacterium* 9 1 43BFAA, *Streptococcus Mitis oralis pneumoniae* and other bacteria (these species are enriched in healthy controls.) showed a negative correlation with clinical data. The abundance of *Bacteroides eggerthii* species was higher in shift workers. Participants with circadian rhythm disorder are more tired and have a poorer ability to overcome sleepiness, which is also related to the enrichment of *Bacteroides eggerthii*. *Streptococcus mitis oralis pneumoniae* and *Eubacterium eligens* are positively related to people’s fatigue and whether they can overcome sleepiness. *Bacteroides Massiliensis* is positively correlated with gastrointestinal symptoms, depression, insomnia, fatigue, and other symptoms, among which the correlation with the degree of insomnia is the strongest.

To understand the functional consequences of the microbial changes in shift workers, we depicted pathways in all metagenomes (**Figure 2F**). There were 19 different metabolic pathways in the two groups. Among the differential metabolic pathways, 18 were significantly increased in shift workers. Phenylacetate degradation I (aerobic) represented the most significantly enhanced functional pathway in shift workers. Allantoin degradation (anaerobic), creatinine degradation, pyruvate fermentation to butanoate, superpathway of Clostridium acetobutylicum acidogenic fermentation, etc. presenting in participants with circadian rhythm disorder, showed enhancement. In the healthy population, the C4 photosynthetic carbon assimilation cycle showed enhancement. Indicating great differences in metabolic pathways between the two groups, which possibly explains the difference in metabolites between the two groups to some extent. In our research findings, multiple key metabolites and bacteria associated with the allantoin degradation IV (anaerobic) pathway showed simultaneous enrichment. *Eubacterium eligens* associated with this pathway can degrade and recover allantoin in an anaerobic manner, which is an important way to ensure the metabolic balance of the human body. Host intestinal microorganisms can also ferment pyruvate to produce and acetyl coenzyme A (Bogorad et al., 2013). Butyric acid can be formed by condensation of two acetyl coenzymes A and subsequent reduction to butyryl coenzyme A. The activation of the pyruvate fermentation to butyrate pathway suggests that butyrate production is functionally active.

### 3.3 Metabolome alterations between the circadian rhythm disorder population and healthy controls

**Figure 3:**
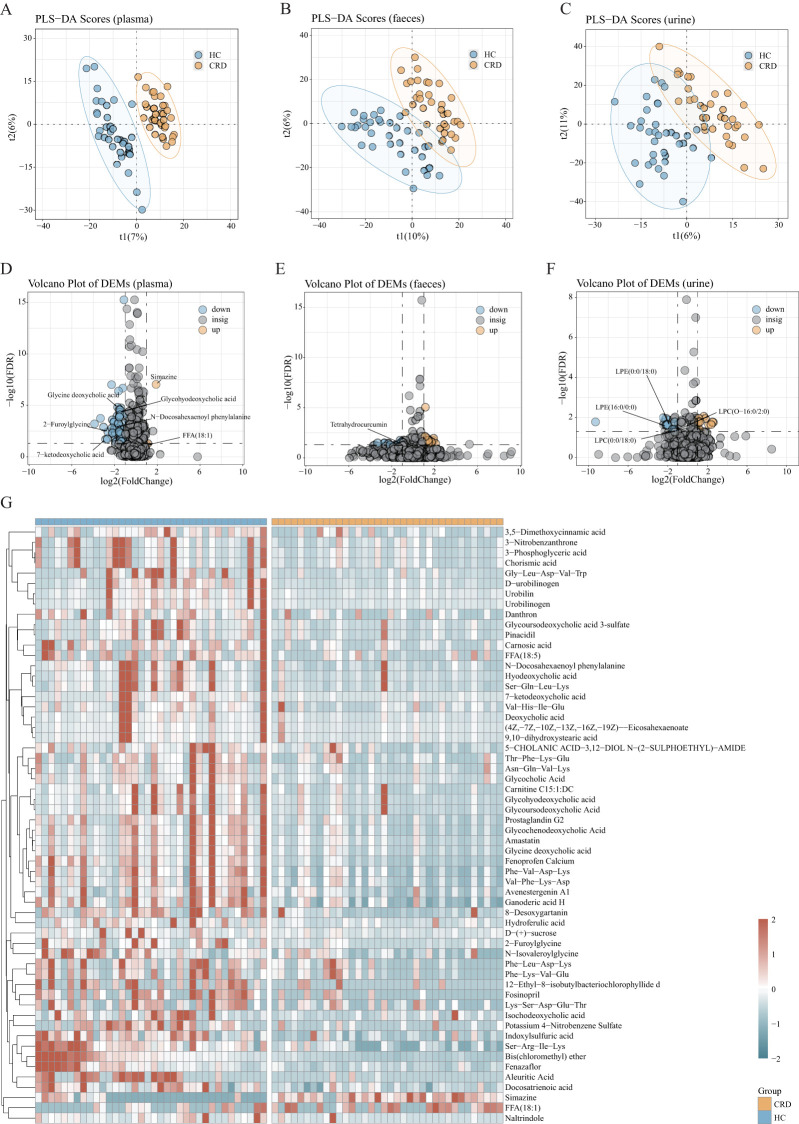
Differentially abundant metabolites. **(A-C)** PLS-DA score plots based on metabolomic profiles of 32 shift workers and 32 healthy controls form the discovery cohort in both positive and negative ion modes. The stool samples were 31 participants with circadian rhythm disorder and 32 controls. **(D-F)** The differential metabolites were screened according to the model obtained in **(A-C)**. Volcano plots depicting the changes in representation for the 1759 plasma metabolites identified in our shift worker and healthy controls individuals. There are 2943 fecal differential metabolites and 3036 urine differential metabolites. **(G)** Heat map of the 58 significantly different metabolites (plasma) across CRD and healthy controls. Most of the plasma differential metabolites were enriched in the healthy controls.

Metabolomics testing was performed on all plasma, urine, and fecal samples. Through PLS-DA (**Figures 3A–C**), we found considerable differences in metabolomics between participants with circadian rhythm disorders and healthy controls. A total of 1759 metabolites were captured in plasma, 2943 in feces, and 3036 in urine. Through Volcano Plot of DEMs (**Figures 3D–F**), we found that 58 kinds of plasma metabolites (fdr<0.05 vip>=1, fc>2 | fc<1/2) had significant changes in participants with circadian rhythm disorder; there were 38 changes in fecal metabolites and 29 changes in urine metabolites. The most significant difference was in blood metabolomics. The following detailed analysis is mainly based on plasma metabonomics.

There was a total of 58 plasma differentially abundant metabolites between the two groups (**Figure 3G**), of which 2 were enriched in the CRD and 56 were enriched in the healthy controls. Many differentially abundant metabolites were concentrated in healthy controls, and bile acid-related metabolites were the most abundant. For example, 7-ketodeoxycholic acid, glycine deoxycholic acid, glycohyodeoxycholic acid, etc. In addition to some differentially abundant metabolites caused by environmental and dietary factors, there are also some fatty acid-related metabolites, such as 2-furoylglycine and N-isovaleroylglycine. Metabolites with signal functions, such as N-docosahexaenoyl phenylalanine can be synthesized both endogenously and by gut microbiota. These metabolites have a variety of signaling functions in physiology, including cardiovascular activity, metabolic homeostasis, memory, cognition, pain, and motor control. There are only two kinds of differentially abundant metabolites enriched in participants with circadian rhythm disorders: free fatty acids (FFAs) and human exposure.

We screened 38 different metabolites (**Supplementary Figure 1A**) in fecal samples from the two groups, 24 of which were enriched in participants with circadian rhythm disorders. Benzene and substituted derivatives, as well as organic acid and its derivatives, are the main metabolites enriched in participants with circadian rhythm disorders. Most of them are metabolites of food and drug contact, as well as some metabolites that may come from environmental and occupational contact. Tetrahydrocurcumin was enriched in the normal population. The Human Metabolome Database (HMDB) showed that it was the product of CUR oxidation by gut microbiota.

There were fewer urine samples observed in differentially abundant metabolites than in the other two metabolites. A total of 29 differentially abundant metabolites in urine (**Supplementary Figure 1B**) were screened out. Few amounts of small peptides without obvious biological significance, as well as some lysophospholipids, hormones and hormone-related compounds, heterocyclic compounds, etc, were observed. Lysophospholipids were significantly increased in the urine of healthy controls, including lysophosphatidylcholine and lysophosphatidylethanolamine.

### 3.4 Associations between plasma metabolites, questionnaire factors and gut microbiota

Based on the above, we learned that there were differences in the gut microbiota and large differences in metabolites in feces, plasma, and urine between participants with circadian rhythm disorders and healthy participants. However, these differences result in joint action of multiple factors, not only of the gut microbiota. This study explored whether the physical and mental health of participants with circadian rhythm disorders is related to gut microbiota and metabolites. Therefore, we included the collected questionnaire factors in the association analysis.

Our use of multiomics combined with the measured data allowed us to identify the interacting relationship between differentially expressed microorganisms and metabolic characteristics in participants with circadian rhythm disorders. In the following, the correlation data of species, metabolites and clinical data showed moderate correlation (|r|>0.3, p<0.05). We mainly focus on the data related among the three groups.

**Figure 4:**
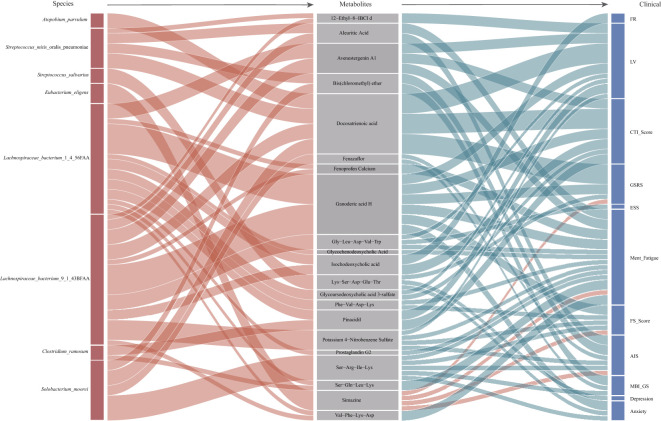
Mediation linkages among species, plasma metabolites, and questionnaire factors. The Sankey plot indicates the mediation relationships among differentially abundant species, plasma metabolites, and questionnaire factors. The ribbons were colored according to the correlation between various factors, with red indicates positive correlation and blue indicates negative correlation. ^a^IBCI, isobutylbacteriochlorophyllide.

There were 34 significant correlations between 21 blood metabolites and 8 fecal microorganisms (**Figure 4**, Left half of figure). Most of the correlations focused on *L. bacterium*. Both *Lachnospiraceae bacteria* (*L. bacterium* 1 4 56FAA and *L. bacterium* 9 1 43BFAA) which showed 21 associations with 16 blood differentially abundant metabolites. Most of these correlations are positive, which means that the gut microbiota and metabolism are in a state of common promotion. A large class of metabolites related to bacteria such as *L. bacterium* and *Clostridium ramosum* are bile acid-related metabolites. Bile acid-related metabolites such as glycohyodeoxycholic acid and glycocholic acid can reflect human liver function (Evangelakos et al., 2021) to some extent. Other gut microbiota, such as *Clostridium ramosum* and *Streptococcus mitis oralis pneumoniae*, also accounted for a large proportion of the bacteria collected in the correlation analysis.

Then, we analyzed the association of clinical questionnaire factors with plasma differentially abundant metabolites. Correlation analysis showed that the changes in plasma differentially abundant metabolites in the participants with circadian rhythm disorder had a certain correlation with the questionnaire factors.

At present, there are 51 associations between 21 different blood metabolites and 11 questionnaire factors in participants with dysrhythmia (**Figure 4**, Right half of figure). Bile acid-related metabolites(Glycochenodeoxycholic Acid, Isochodeoxycholic acid, Glycoursodeoxycholic acid 3-sulfate) were negative correlation with five questionnaire factors. In the correlation between plasma metabolites and species, there was also a positive correlation between bile acid-related metabolites and *L. bacterium*. They are a large group of blood differentially abundant metabolites. Perhaps for this phenomenon, we can understand that the clinical symptoms of circadian rhythm disorder and metabolic disorders are associated with *L. bacterium*. Docosatrienoic acid, belongs to the class of organic compounds known as very long-chain fatty acids. It is negatively correlated with three questionnaire factors and positively correlated with multiple gut microbiota (including *L. bacterium* 9 1 43BFAA). Perhaps the increase in these two *L. bacterium* can alleviate the symptoms caused by rhythm disorder.

In the correlation analysis between fecal differentially abundant metabolites and questionnaire factors (**Supplementary Figure 2A**), we also noticed that tetrahydro curcumin was negatively correlated with two questionnaire factors and positively correlated with two kinds of *L. bacterium* (9 1 43BFAA and 1 4 56FAA). A small amount of correlation analysis data (**Supplementary Figure 2B**) of urine was not described here.

In general, most of our species and metabolites were positively correlated, while most of the differentially abundant metabolites were negatively correlated with clinical data. It’s possible that some changes in metabolites corresponding to different gut microbiota can affect human clinical manifestations. In our correlation analysis, two kinds of *L. bacterium* were associated with a variety of different metabolites and questionnaire factors.

## 4 Discussion

This study reported an integrated analysis of the microbiome and fecal/plasma/urine metabolome of shift workers. We observed that the gut microbiota and various metabolomics of participants with circadian rhythm disorder had certain changes compared with healthy controls. Metabolomics testing showed that changes in plasma metabolomics were more pronounced than changes in fecal and urinary metabolomics. Although we did not observe how significant changes exist in the gut microbiota composition of participants with circadian rhythm disorders, we still screened 19 different species with different colonization conditions and 19 metabolic pathways related to these different species. Furthermore, our correlation analysis results showed that some species and metabolites were also closely related to the degree of clinical symptoms in participants with circadian rhythm disorders. These significant species and metabolites may be able to intervene and alleviate the damage caused by circadian rhythm.

Fecal metagene data showed differences in multiple metabolic pathways. Among these differential metabolic pathways, the phenylacetate degradation pathway widely exists in the field of bacteria (Jiao et al., 2022). Changes in the phenylacetate degradation pathway are related to the tolerance of bacteria to antibiotics and hydrogen peroxide stimulation (Hooppaw et al., 2022). In *Escherichia coli* and several *Pseudomonas* sp*ecies*, this metabolic pathway includes both aerobic and anaerobic pathways (Jiao et al., 2022; Hernández-Rocamora et al., 2024). In the aerobic pathway, the accumulation of early products (ring-1,2-epoxide and its phenolic breakdown product 2-hydroxyphenylacetate) of phenylacetic acid degradation may have toxic effects on the host (Jiao et al., 2022). In a variety of infectious diseases caused by *Acinetobacter baumannii*, disrupting the catabolism of PAA will interfere with its adaptability, leading to the reduction of its resistance to antibiotics and hydrogen peroxide (Hooppaw et al., 2022). The activity of aerobic pathways in our research results reveals an increased likelihood of disease in individuals with circadian rhythm disorders due to microbial metabolites.

Allantoin degradation IV (anaerobic) is a functional pathway. Some organisms, including *Escherichia coli* K12 and *Delftia acidovorans* (Caspi et al., 2018), can utilize allantoin as a nitrogen source under anaerobic conditions. It is well known that allantoin is the product of uric acid oxidation after purine metabolism (Keenan, 2020), and maintaining the steady state of purine metabolism is very important for the human body (Furuhashi, 2020; He et al., 2022). However, we only observed an increase in the catabolic pathway of allantoin, a product of uric acid oxidative stress, that’s not directly related to the process of purine metabolism. Pyruvate fermentation to butanoate is a subpathway of the superpathway of *Clostridium acetobutylicum* acidogenic fermentation. Butyrate is a very popular microbial metabolite in various studies. It can help maintain the health of intestinal cells, reduce the occurrence of diseases, and has important research value in antitumor (Che et al., 2023; Zhu et al., 2023), metabolic homeostasis (Li et al., 2023), anti-inflammatory (Wang et al., 2022), and other aspects. Although we observed the activity of the butyric acid pathway here, we did not observe changes in butyric acid-related metabolites.

The aforementioned functional metabolic pathways of microbiota indicate that the observed aspects on gut microbiota have influenced the metabolic regulation of participants with circadian rhythm disorders. However, due to limited supporting data on metabolomic, we only focused on gut microbiota metabolism and indicated that the differential microbiota has a certain function for participants with circadian rhythm.

Bile acid-related metabolites represent a major class of metabolites that were significantly altered in participants with circadian rhythm disorders. Compared with the normal population, it is deficient in participants with circadian rhythm disorders. Bile acid metabolism in the gut microbiota can be associated with nonalcoholic fatty liver disease, irritable bowel syndrome, colorectal cancer, neuroinflammation, and early aging (Fogelson et al., 2023). Here, the gut microbiota of the host can affect bile acids, which in turn can shape the composition of the host-microbe (Collins et al., 2022). Reducing the level of bile acids can reduce the absorption of lipids (Ding et al., 2021). We also observed a decrease in fatty acid metabolites (2-furoylglycine) and an increase in FFAs in the plasma metabolomics which results in participants with circadian rhythm disorders. This can confirm the role of bile acids in the regulation of lipid metabolism, and it also indicates that the function of participants with circadian rhythm disorder for fatty acid metabolism is weakened.

We know that shift work will affect people’s physical and mental states, which has been confirmed in our scale data. Linking metabolomic and microbiological data, we found that many species and metabolites were associated with these clinical manifestations. For example, in our correlation analysis, the interaction between *L. bacterium* and bile acid-related metabolites and the negative correlation between bile acid-related metabolites and human fatigue and other score data. Perhaps we can think that the gut microbiota and related metabolites of participants with circadian rhythm disorder can affect the clinical manifestations. This may be supported by some gut brain axis-related studies (Margolis et al., 2021). Further research is needed on this matter. In the joint analysis of metabolic microbiota diseases, the differentially abundant metabolites and species were analyzed more deeply (Han et al., 2021).

### 4.1 Conclusion

In summary, our research shows that participants with circadian rhythm disorder will have changes in gut microbiota. The activity of some functional metabolic pathways of microbiota (phenylacetic acid degradation) may lead to the accumulation of harmful metabolites and affect human health. The joint analysis of microbiota-metabolite-clinical data showed that the gut differential microbiota promoted the production of most of the plasma differentially abundant metabolites, and the production of these metabolites was negatively correlated with our clinical data. The association of gut microbiota metabolite scale data suggests a new and potential way to intervene in circadian rhythm disorder. It may be possible to regulate the physical and mental discomfort caused by circadian rhythm disorder through the intervention of personalized species or metabolites.

We acknowledge that our study has certain limitations, such as the narrow occupational range of the participants; inability to make causal inferences due to the cross-sectional study design. In this study, we did not perform validation experiments for the observed differential pathways, metabolites, or species. Our study integrates multi-omics measurements, with the dataset encompassing detailed participant clinical data, metagenomics, and metabolomics data. We hope that these publicly available research findings and datasets will facilitate future validation in broader populations and contribute to mechanistic explorations.

## Data Availability

The datasets presented in this study can be found in online repositories. The names of the repository/repositories and accession number(s) can be found below: China National GeneBank DataBase, CNP0004943. https://db.cngb.org/.
