# Supplementary material for: Multi-omics assessment of gut microbiota in circadian rhythm disorders: a cross-sectional clinical study
Source: Front Cell Infect Microbiol. 2025 Mar 27;15:1524987. doi: 10.3389/fcimb.2025.1524987 (PMC11983646; doi:10.3389/fcimb.2025.1524987)
Supplement: Supplementary file 1 [file DataSheet1.docx]

Supplementary Material

## Supplementary Figures


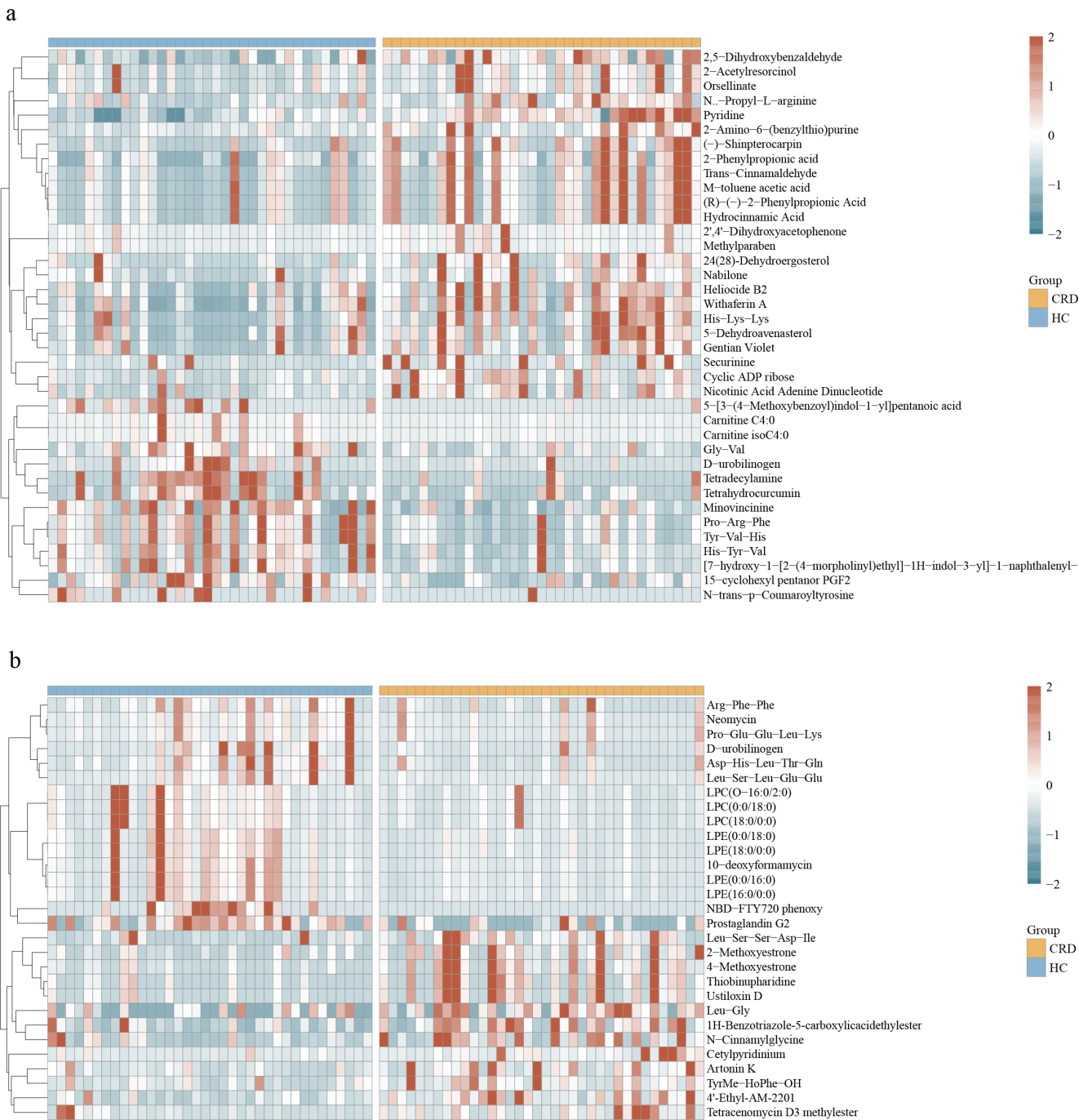


**Supplementary Figure1.** Differentially abundant feces and urine metabolites. (A)Heat map of the 38 significantly different metabolites (feces) across CRD and healthy controls. (B) shows urine (29 significantly different metabolites).


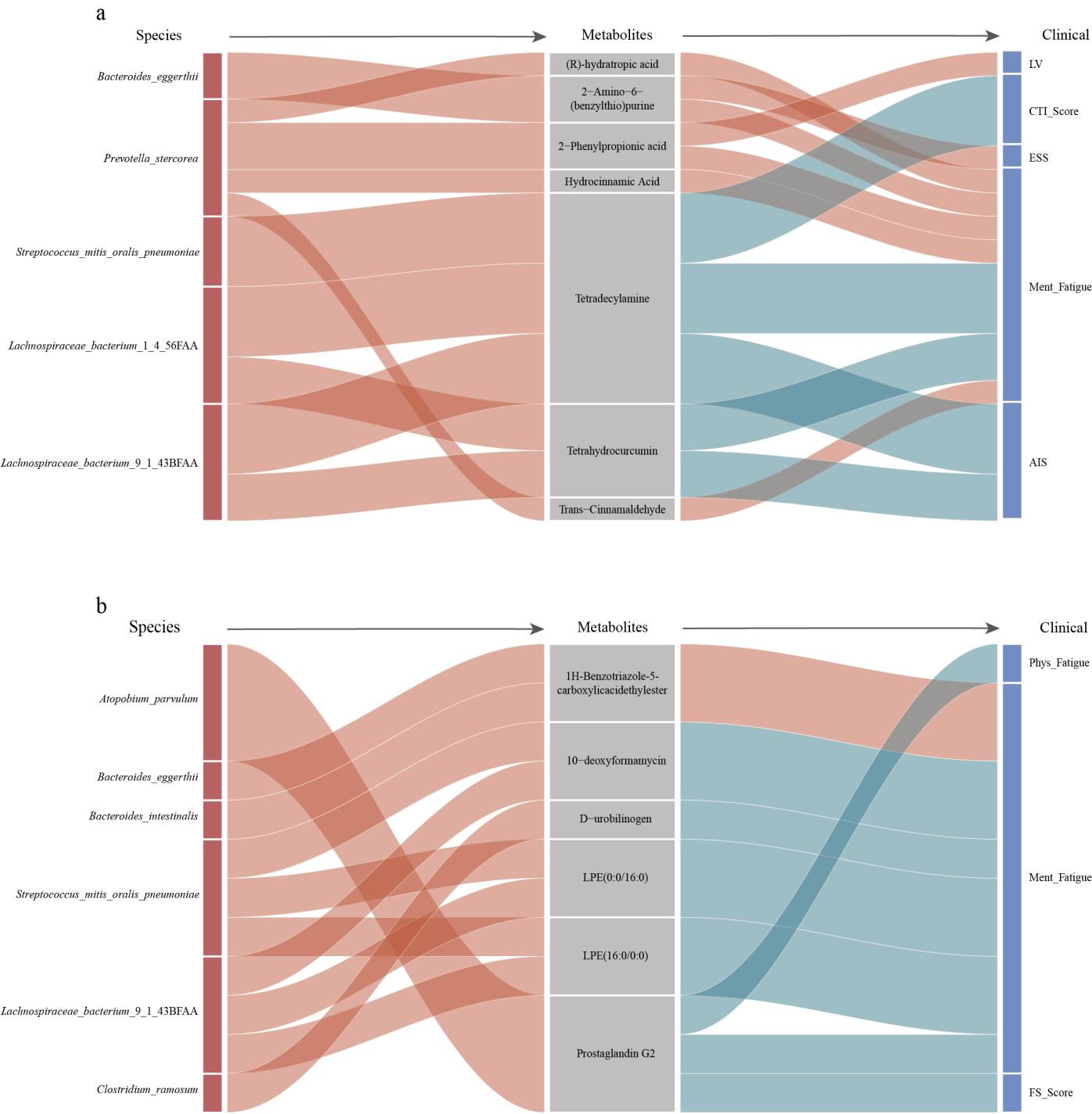


**Supplementary Figure2.** Mediation linkages among species, feces/urine metabolites, and questionnaire factors. The Sankey plot indicates the mediation relationships among differentially abundant species, metabolites, and questionnaire factors. (A) shows feces; (B) shows urine.
